# The Comparative Prognostic Value of the Interim PET Deauville Score and ΔSUVmax in Classical Hodgkin Lymphoma: A Real-World Study of Imaging and Inflammatory Markers

**DOI:** 10.3390/jcm15197353

**Published:** 2026-09-22

**Authors:** Sinan Mersin, Hüseyin Yanık, Zehra Pınar Koç, Pınar Pelin Özcan, Gülsüm Ayrık Meşeli, Mahmut Bakır Koyuncu, Pelin Aytan, Ayça Alantaş, Nisa Ilgım Bolat, Ferhat Akad, Aycan Erkenekli, Esin Oğuz Kozan, Enver Tekin, Eyüp Naci Tiftik

**Affiliations:** 1 Division of Hematology, Department of Internal Medicine, Faculty of Medicine, Mersin University, 33110 Mersin, Türkiye; 2 Department of Information Systems and Technologies, School of Applied Technology and Management, Mersin University, 33730 Mersin, Türkiye; 3 Department of Nuclear Medicine, Faculty of Medicine, Mersin University, 33343 Mersin, Türkiye; 4 Faculty of Medicine, Mersin University, 33343 Mersin, Türkiye; 5 Department of Hematology, Adıyaman Training and Research Hospital, 02200 Adıyaman, Türkiye

**Keywords:** Hodgkin lymphoma, interim PET/CT, Deauville score, ΔSUVmax, lymphocyte-to-monocyte ratio

## Abstract

**Background:** Interim [18F]FDG-PET/CT with the Deauville score (DS) is central to response assessment in classical Hodgkin lymphoma (cHL). Quantitative ΔSUVmax and the lymphocyte-to-monocyte ratio (LMR) may refine the DS, but ΔSUVmax thresholds vary across cohorts and have rarely been compared. We tested published ΔSUVmax thresholds and compared the value added by ΔSUVmax and the LMR to the DS for progression-free survival (PFS). **Methods:** We retrospectively analyzed 78 adults with cHL with paired baseline and interim PET/CT. Outcomes were measured from the interim scan using Kaplan–Meier analysis, Cox regression, and Harrell’s C-index with bootstrap validation. **Results:** Over 47 months’ median follow-up, 26 PFS events occurred. Higher published ΔSUVmax thresholds (83–88%) did not separate PFS, whereas 66% did; ΔSUVmax added no value to the DS. DS positivity predicted worse PFS (HR 2.26, *p* = 0.040). Lower LMR added value to the DS (HR 0.45, *p* = 0.006) but overlapped with the International Prognostic Score (IPS; ρ = −0.51) without surpassing it. A DS-plus-IPS model stratified three risk groups (5-year PFS 83%/60%/24%) and outperformed DS-plus-LMR (C-index 0.73 vs. 0.66). **Conclusions:** Published ΔSUVmax thresholds were not reproducible, and ΔSUVmax did not improve on the DS. The LMR added independent information but did not surpass the IPS; a simple DS-plus-IPS model provided effective, low-cost risk stratification.

## 1. Introduction

Classical Hodgkin lymphoma (cHL) is among the most curable hematologic malignancies, with long-term survival of approximately 80–90% following contemporary first-line therapy [1]. It typically presents with painless lymphadenopathy and, in a subset, systemic ‘B symptoms’ (fever, night sweats, and unexplained weight loss), and its diagnosis rests on the histopathological identification of Hodgkin/Reed–Sternberg cells expressing CD30 and CD15 within a reactive inflammatory background [1]. Despite these outcomes, primary refractory disease or relapse still occurs in approximately 10–15% of early-stage and 15–30% of advanced-stage patients, and accurate early risk stratification is essential to identify those who may benefit from treatment adaptation [2,3]. Interim [18F]FDG-PET/CT performed after two chemotherapy cycles (PET-2) has become the strongest early predictor of outcome and, in the study by Gallamini et al., was prognostically superior to the International Prognostic Score (IPS) in advanced-stage disease [4]. Interim [18F]FDG-PET/CT is a metabolic imaging scan performed during first-line treatment to assess early response by comparison with the pretreatment (baseline) scan [4,5]. In the RATHL trial, for example, patients with a negative PET-2 had a 3-year progression-free survival (PFS) of 85.7%, compared with 67.5% for those with a positive PET-2 [6]. Interim response is interpreted using the visual Deauville 5-point score (DS) within the Lugano classification, in which a score of 1–3 denotes a favorable (negative) and 4–5 an unfavorable (positive) result [5,7].

Although practical and widely adopted, the DS is an ordinal, visual metric subject to interobserver variability, particularly for residual uptake near the mediastinal blood pool and liver reference thresholds [5]. Quantitative assessment of the change in maximum standardized uptake value (ΔSUVmax) between the baseline and interim scans has been proposed as a more objective alternative and, in some cohorts, reported to be superior to visual analysis for predicting early outcomes [8]. However, the ΔSUVmax cutoff associated with prognosis has varied considerably across studies—approximately 71%, 83%, 88%, and 93% in different HL series—raising concerns about the reproducibility and transportability of any single threshold [8,9,10,11]. Moreover, although much of the quantitative-PET evidence derives from diffuse large B-cell lymphoma, the available cHL-specific data—principally the series of Rossi et al. [8] and Pinczés et al. [10]—are limited and have produced heterogeneous thresholds, a gap that the present study addresses.

In parallel, host- and microenvironment-derived markers have attracted growing interest. In cHL, malignant Hodgkin/Reed–Sternberg cells constitute only a minor fraction of the tumor, embedded within an abundant reactive infiltrate, and a high content of CD68+ tumor-associated macrophages predicts an inferior outcome [12]. The peripheral-blood lymphocyte-to-monocyte ratio (LMR) is a simple, inexpensive index that reflects the host systemic inflammatory and immune milieu—an indirect marker rather than a direct surrogate for tumor-associated macrophage content—and a low LMR at diagnosis has been repeatedly associated with worse survival in cHL [13,14]. Combining the LMR with interim PET has been reported to provide independent and incremental prognostic value, with the two markers reinforcing each other, and inflammatory-based prognostic models for HL continue to emerge [15,16,17].

Thus, a quantitative imaging marker (ΔSUVmax) and a host inflammatory marker (LMR) have each been proposed to refine interim, DS-based assessment. To our knowledge, however, these two candidate refinements have not been directly compared within a single cohort, and the reproducibility of published interim ΔSUVmax thresholds in unselected, real-world populations remains unclear.

In a real-world, single-center cohort of adults with cHL, we therefore aimed to (i) test whether previously published interim ΔSUVmax thresholds are reproducible; (ii) compare the incremental prognostic value for PFS of ΔSUVmax and of the LMR when added to the visual DS; and (iii) explore a simple, clinically applicable risk model combining interim PET response with baseline clinical risk.

## 2. Materials and Methods

### 2.1. Study Design and Patients

We performed a retrospective, single-center, real-world cohort study at Mersin University Faculty of Medicine, Department of Hematology (Mersin, Türkiye). We retrospectively identified consecutive adults (≥18 years) with histologically confirmed classical Hodgkin lymphoma diagnosed and treated at our center from 2014 onward (*n* = 195). Of these, 96 had both a baseline and an interim [18F]FDG-PET/CT with complete follow-up at our center. The remaining 99 patients did not have complete follow-up at our institution: as a tertiary referral center, we frequently receive patients who either present at relapse after having received first-line therapy elsewhere or elect to continue first-line treatment at another institution, and a smaller number had incomplete follow-up data at our center; in all such cases, paired baseline and interim PET/CT with subsequent clinical follow-up were not available at our institution. We further excluded 5 patients with nodular lymphocyte-predominant Hodgkin lymphoma and 13 patients whose baseline or interim PET could not be evaluated for quantitative analysis owing to poor image quality or inaccessible source (raw) image data that precluded reliable SUVmax measurement, yielding a final analysis cohort of 78 patients (Figure 1). The study was conducted in accordance with the Declaration of Helsinki and approved by the Health Sciences Research Ethics Committee of Mersin University (approval no. 2026-419, date 8 July 2026); given the retrospective design, the requirement for written informed consent was waived, and all data were anonymized before analysis.

### 2.2. PET/CT Acquisition and Interpretation

[18F]FDG-PET/CT was performed at baseline and after two cycles of first-line chemotherapy (interim PET, PET-2) using a dedicated PET/CT scanner (Siemens Biograph mCT 20; Siemens Healthineers, Erlangen, Germany). Images were acquired in the craniocaudal direction from the vertex to the mid-thigh (511 keV; field of view, 780 mm; 200 × 200 matrix; TrueX and Time-of-Flight [UltraHD-PET] reconstruction; 21 subsets; 2 iterations). A low-dose CT scan (slice thickness, 3 mm; 13–416 mAs; 120 kVp) was performed for attenuation correction. All baseline and interim scans were interpreted by a single experienced nuclear medicine physician (Z.P.K.) who was blinded to prior results and to clinical outcome. Interim response was assessed visually using the Deauville 5-point score (DS), with the mediastinal blood pool and liver as reference; DS 1–3 was classified as negative and DS 4–5 as positive [5,7]. For quantitative assessment, the same physician measured the SUVmax of the single most FDG-avid lesion on the baseline scan and of the most FDG-avid residual lesion on the interim scan—the two reference lesions were not required to be anatomically identical, consistent with published ΔSUVmax methodology [18]—using OsiriX MD software (version 14.01; Pixmeo, Geneva, Switzerland) on a workstation, and ΔSUVmax (%) was calculated as (baseline SUVmax − interim SUVmax)/baseline SUVmax × 100. In patients without measurable residual metabolic activity on interim PET, the interim SUV was set to 0 and ΔSUVmax to 100%, consistent with prior reports [8,10]. The reference lesion and the mediastinal and hepatic reference SUVmax values were recorded. As all reads were performed by the same physician, but the visual DS and the quantitative SUVmax measurements were not independently determined; interobserver reproducibility could therefore not be assessed.

### 2.3. Clinical and Laboratory Variables

Disease was staged using the Ann Arbor system with Cotswolds modifications, incorporating [18F]FDG-PET/CT findings, in accordance with the Lugano classification [7]; stages I–II were classified as early-stage and stages III–IV as advanced-stage disease. Baseline demographic, clinical, and laboratory data were collected at diagnosis, including Ann Arbor stage, the presence of B symptoms, bulky mediastinal disease, extranodal involvement, and histological subtype. The International Prognostic Score (IPS) was calculated from its seven original components (serum albumin < 4 g/dL, hemoglobin < 10.5 g/dL, male sex, age ≥ 45 years, stage IV disease, leukocytosis ≥ 15 × 10^9^/L, and lymphocytopenia < 0.6 × 10^9^/L or <8% of the white-cell count) [19]. From the peripheral blood counts obtained at diagnosis, the lymphocyte-to-monocyte ratio (LMR), neutrophil-to-lymphocyte ratio (NLR), and platelet-to-lymphocyte ratio (PLR) were calculated from the corresponding absolute counts. The first-line chemotherapy regimen and the end-of-treatment response were recorded. During follow-up, 26 PFS events (21 progressions or relapses and 5 deaths without documented progression) and 10 deaths (overall-survival events) were observed.

### 2.4. Endpoints

The primary endpoint was progression-free survival (PFS), defined as the time from the interim PET/CT scan to disease progression, relapse, or death from any cause, whichever occurred first. Overall survival (OS) was defined as the time from the interim PET/CT scan to death from any cause. Patients without an event were censored at the date of last follow-up. This landmark definition, anchored at the interim scan, was chosen to match prior interim-PET studies and to avoid immortal-time bias [10,18]. Because both a baseline and an interim scan were required for inclusion, patients who progressed or died before the interim PET/CT were, by definition, not part of the analyzed cohort. In our series, 4 patients died before an interim PET/CT could be performed—1 shortly after diagnosis before treatment initiation and 3 during first-line treatment—and were therefore not part of the analyzed cohort; no patient in the analyzed cohort progressed before the interim scan. Primary refractory disease was defined as progression during first-line therapy or within 3 months of its completion, and relapse as recurrence after a prior complete response.

### 2.5. Statistical Analysis

Continuous variables were summarized as median [interquartile range, IQR] and categorical variables as counts (percentages); normality was assessed with the Shapiro–Wilk test. Between-group comparisons used the Mann–Whitney U test or the Student *t*-test for continuous variables, and the χ^2^ or Fisher exact test for categorical variables, as appropriate. Survival was estimated using the Kaplan–Meier method and compared with the log-rank test; median follow-up was estimated by the reverse Kaplan–Meier method. Associations with PFS and OS were assessed using Cox proportional-hazards regression, with hazard ratios (HR) and 95% confidence intervals (CI); the proportional-hazards assumption was verified using scaled Schoenfeld residuals. Discrimination was quantified with Harrell’s concordance index (C-index), and internal validity was assessed by bootstrap optimism correction (up to 2000 resamples). Previously published ΔSUVmax thresholds (66%, 71%, 83%, 88%, and 93%) were evaluated as pre-specified cutoffs [8,9,10,11,18,20]. Data-derived optimal thresholds for ΔSUVmax and the LMR were identified using maximally selected log-rank statistics, with permutation-based correction of the resulting *p*-value and bootstrap assessment of cutpoint stability to account for the multiplicity inherent in cutpoint optimization. The incremental value of a marker over the DS was tested using nested Cox models and the likelihood-ratio test. For the inflammatory markers examined jointly, the Benjamini–Hochberg false discovery rate was applied. Multivariable models were limited to a maximum of two covariates given the number of events, to maintain an events-per-variable ratio of approximately ≥10. Analyses were pre-specified as confirmatory (reproducibility of published ΔSUVmax thresholds; the DS; continuous ΔSUVmax) or exploratory (data-derived thresholds; the DS-plus-IPS risk model; subgroup and interaction analyses), and exploratory findings are labeled accordingly. A two-sided *p* < 0.05 was considered statistically significant. Analyses were performed using Python (version 3.12.3) with the lifelines (v0.30.3), SciPy (v1.17.1), statsmodels (v0.14.6), NumPy (v2.4.4), and pandas (v2.3.3) packages.

## 3. Results

### 3.1. Patient Characteristics

Seventy-eight patients with classical Hodgkin lymphoma were included. The median age was 40.5 years (IQR 26.5–52.0), and 54 (69%) were male. Mixed cellularity was the most common histological subtype (48 patients, 62%), followed by nodular sclerosis (26, 33%) and lymphocyte-rich (4, 5%). Advanced-stage (Ann Arbor III–IV) disease was present in 45 patients (58%), B symptoms in 46 (59%), and bulky mediastinal disease in 25 (32%); the median IPS was 3 (IQR 1–4). Most patients (63, 81%) received ABVD-based first-line chemotherapy. Interim PET was positive (DS 4–5) in 22 patients (28%). Baseline clinical, laboratory, and imaging characteristics are summarized in Table 1. Over a median follow-up of 47 months (reverse Kaplan–Meier), 26 PFS events and 10 deaths occurred; the estimated 5-year PFS and OS for the whole cohort were 62.5% (95% CI 48–74) and 85.8%, respectively.

### 3.2. Reproducibility of Published Interim ΔSUVmax Thresholds

We first evaluated, as pre-specified cutoffs, the ΔSUVmax thresholds previously reported in cHL. The higher thresholds did not discriminate PFS: for ΔSUVmax > 88% [10], the 5-year PFS was 59.3% versus 63.1% (log-rank *p* = 0.49), and for >83% [9], 67.6% versus 56.4% (*p* = 0.39). In contrast, lower thresholds retained prognostic value: a cutoff of >66%—the classical value derived in diffuse large B-cell lymphoma [20] and discussed in the Menton consensus [18]—separated PFS (66.3% versus 45.5%; *p* = 0.033; HR 2.43, 95% CI 1.05–5.63), with a similar trend at >71% [8] (*p* = 0.062). A data-derived optimal threshold fell at approximately 64–66%; however, after permutation-based correction for cutpoint selection, this was only of borderline significance (corrected *p* = 0.056), and the cutpoint was unstable on bootstrap resampling (median 64%, 95% interval 52–90%). Thus, no single published ΔSUVmax threshold was transportable to our cohort, and the discriminating threshold, where present, was lower than the higher HL-specific values reported recently (Table 2).

### 3.3. Deauville Score, Continuous ΔSUVmax, and Their Comparison

A positive interim DS (4–5) was associated with worse PFS (5-year PFS 51.0% versus 66.9%; HR 2.26, 95% CI 1.04–4.94; *p* = 0.040). As a continuous variable, ΔSUVmax was weakly associated with PFS (HR 0.86 per 10% increase, 95% CI 0.75–0.98; *p* = 0.024), but its discrimination was poor (C-index 0.53) and inferior to that of the DS (C-index 0.60 for DS positivity and 0.69 for the ordinal DS). In nested Cox models, ΔSUVmax added no prognostic value to the DS (likelihood-ratio *p* = 0.21). The proportional-hazards assumption held for both markers (Schoenfeld *p* > 0.05). The dissociation between a statistically significant association and near-chance discrimination (C-index 0.53) indicates that continuous ΔSUVmax has limited clinical predictive utility at the individual-patient level.

### 3.4. Lymphocyte-to-Monocyte Ratio

A lower LMR at diagnosis predicted worse PFS (HR 0.45 per log-unit, 95% CI 0.26–0.80; *p* = 0.006; C-index 0.65) and, unlike ΔSUVmax, added prognostic value to the DS (likelihood-ratio *p* = 0.013), increasing the C-index from 0.60 to 0.68. For descriptive purposes, a data-derived cutoff of LMR ≤ 1.82 defined a high-risk group (5-year PFS 47.0% versus 71.6%; HR 3.13, 95% CI 1.43–6.84; *p* = 0.004; permutation-corrected *p* = 0.033); however, this exploratory threshold was derived in a single center, had a wide bootstrap confidence interval (2.5–97.5th percentile, 1.07–3.76), and requires external validation. All inferential analyses of the LMR were therefore based on the continuous (log-transformed) variable. The prognostic effect of the LMR was independent of the DS and of stage but not of the IPS, with which it was moderately correlated (Spearman ρ = −0.51). Among the other inflammatory indices, a higher NLR was associated with worse PFS on univariable analysis (HR 1.81, *p* = 0.049) but did not survive correction for multiple testing (FDR-adjusted *p* = 0.073) and added nothing to either the DS (*p* = 0.12) or the DS-plus-LMR model (*p* = 0.88); the PLR was not prognostic (*p* = 0.43). Overall survival analyses were underpowered given only 10 deaths; neither the DS (HR 1.69, 95% CI 0.47–6.03; *p* = 0.42) nor the LMR (HR 0.52, 95% CI 0.23–1.18; *p* = 0.12) reached significance for OS, which was therefore treated as a secondary, exploratory endpoint.

### 3.5. A Combined Deauville-Plus-IPS Risk Model

Because the DS and the IPS captured complementary imaging and clinical information, we combined them into a three-tier model (low risk, DS-negative and IPS < 3; high risk, DS-positive and IPS ≥ 3; intermediate, all others). The IPS threshold of ≥3 was chosen as an established marker of higher-risk disease and DS 4–5 as the standard definition of a positive interim scan; combining these pre-defined, routinely used categories avoided data-driven cutoff selection. This model separated three groups with markedly different outcomes: the 5-year PFS was 82.9% (low, n = 30), 60.0% (intermediate, n = 34), and 23.6% (high, n = 14) (log-rank *p* < 0.001). Relative to the low-risk group, the HR was 3.34 (95% CI 1.09–10.25) for intermediate and 11.52 (95% CI 3.37–39.34) for high risk. The optimism-corrected C-index of the model was 0.72; bootstrap optimism was small across all models (0.002–0.016), indicating minimal overfitting (Figure 2). To directly compare the two candidate refinements of the DS, we evaluated discrimination and reclassification for DS + LMR versus DS + IPS (Table 3). The optimism-corrected C-index was higher for DS + IPS (0.73) than for DS + LMR (0.66) or DS alone (0.60). Although the LMR added prognostic value to the DS (likelihood-ratio *p* = 0.013), it did not add value beyond the IPS (*p* = 0.34), and reclassification metrics favored DS + IPS over DS + LMR (NRI −0.52, IDI −0.039). The DS-plus-IPS combination was therefore retained as the primary risk model, with the LMR interpreted as a complementary, low-cost marker rather than a replacement for the IPS.

### 3.6. Exploratory Analyses

Correlations among the markers are shown in Figure 3; ΔSUVmax correlated moderately with the DS (ρ = −0.44) and the LMR with the IPS (ρ = −0.51). In a pre-specified exploratory analysis, the prognostic effect of a positive DS differed by disease stage (DS × stage interaction *p* = 0.048): the DS was strongly prognostic in early-stage disease (n = 33; HR 7.53, 95% CI 1.86–30.42) but not in advanced-stage disease (n = 45; HR 1.17, 95% CI 0.41–3.32) (Figure 4). In an exploratory three-tier evaluation of the DS, patients with a DS of 3 (n = 16) had outcomes similar to those with a DS of 4–5 (n = 22; 5-year PFS 50.5% versus 51.0%; *p* = 0.69) and distinct from a DS of 1–2 (n = 40; 72.0%; *p* = 0.048). No significant interaction was found between the marker effects and histological subtype. Sensitivity analyses censoring the single non-Hodgkin-related death did not materially change the results (DS HR 2.41; LMR HR 0.49).

## 4. Discussion

In this real-world cohort of 78 adults with classical Hodgkin lymphoma, three findings emerged. First, previously published interim ΔSUVmax thresholds were not reproducible: the higher HL-specific cutoffs (83–88%) did not discriminate PFS, and only a lower threshold (66%) retained prognostic value. Second, when compared head-to-head as refinements of the visual Deauville score, quantitative ΔSUVmax added no prognostic value, whereas the host-derived lymphocyte-to-monocyte ratio (LMR) did. Third, a simple model combining the interim DS with the baseline IPS provided clinically meaningful, internally validated risk stratification. To our knowledge, this is the first study to compare the incremental value of ΔSUVmax and the LMR, added to the DS, within a single cHL cohort.

The non-transportability of ΔSUVmax thresholds is the most distinctive observation. Although ΔSUVmax was weakly prognostic as a continuous variable, no single published cutoff separated our patients, and the best-performing threshold (≈66%) coincided with the classic Menton/DLBCL value rather than the higher HL-specific cutoffs reported more recently [18,20]. A likely explanation is case-mix. Our institution is a tertiary referral center, which may partly account for the relatively high proportion of advanced-stage disease and the more frequently PET-positive, higher-risk profile of the cohort (28% DS 4–5, compared with 9% in the derivation cohort [10]) and the correspondingly lower 5-year PFS (62.5% versus ~80%). In such a cohort, the threshold that best separates risk sits lower, and any high, optimized cutoff fails to reproduce. This fragility is inherent to data-derived dichotomization of a continuous variable, as reflected in the unstable bootstrap cutpoint, and cautions against transporting a single ΔSUVmax threshold across centers. These thresholds were therefore not validated in our cohort and require confirmation in larger, multicenter studies.

In contrast, a low LMR at diagnosis independently refined the DS. Although a high content of CD68+ tumor-associated macrophages predicts an inferior outcome in cHL [12], the peripheral LMR is best regarded as an indirect, systemic marker of the host inflammatory and immune milieu—reflecting several host factors rather than serving as a direct biological equivalent of tumor-associated macrophage content. Our results are consistent with, rather than novel relative to, prior reports: the prognostic value of the LMR at diagnosis in cHL is well established [13,14], and combining the LMR with interim PET has previously been shown to strengthen prognostication, with both markers acting as independent, mutually reinforcing factors [15,16]; inflammatory-based prognostic models for HL continue to be proposed [17]. Our contribution is to place the LMR alongside quantitative ΔSUVmax in the same cohort and show that, of the two candidate refinements of the visual DS, the host inflammatory marker—not the quantitative imaging marker—carried added value. We deliberately frame the LMR as complementary rather than independent of established scores: its effect was independent of the DS and of stage but not of the IPS, with which it was moderately correlated, and among the inflammatory indices the NLR added nothing beyond the LMR. The LMR therefore behaves as a single, low-cost parameter that recapitulates part of the composite IPS while adding to the imaging-based response. Formal comparison confirmed this asymmetry: the DS-plus-IPS model discriminated better than a DS-plus-LMR model (optimism-corrected C-index 0.73 versus 0.66), the LMR added no value beyond the IPS, and reclassification metrics favored DS-plus-IPS (Table 3).

From a practical standpoint, the combination that mattered most was the interim imaging response and the baseline clinical score. A three-tier model combining the DS and the IPS separated groups with 5-year PFS of 83%, 60%, and 24% and retained good discrimination after optimism correction (C-index 0.72). Such a model requires only information already available in routine practice—an interim PET report and standard clinical and laboratory variables—and may be particularly useful in resource-aware settings where harmonized quantitative PET is not universally available. This is in keeping with a growing body of real-world, geographically diverse cohorts confirming the central role of interim PET in cHL: a single-center Jordanian series of 245 patients [21] and a real-world Vietnamese cohort of 100 patients [22] both found the interim Deauville score to be a strong, independent predictor of outcome. Notably, in the latter, combining the interim response with baseline clinical risk was particularly discriminating—advanced-stage patients with a low IPS (0–3) had a 3-year PFS of 88.2% versus 42.9% for those with a high IPS (4–7) [22]—mirroring the stratification achieved by our DS-plus-IPS model.

The relative prognostic value of visual versus quantitative interim assessment remains debated. Whereas some studies report that semiquantitative metrics such as ΔSUVmax or the tumor-to-liver ratio refine or outperform the visual DS, particularly for early response [8,11,23], we found that the DS out-discriminated ΔSUVmax and that ΔSUVmax added nothing to it. Because a single reader performed both the visual DS and the SUVmax measurements—which, if anything, would bias the two metrics toward agreement—the absence of added value from ΔSUVmax in our cohort is unlikely to be explained by measurement discordance. Our data therefore add to the evidence that, at least in unselected real-world populations, a single quantitative threshold does not reliably improve on the visual DS.

Several findings were exploratory and are presented as hypothesis-generating. The prognostic effect of a positive DS appeared concentrated in early-stage disease and was absent in advanced-stage disease, a stage-dependence that resonates with the differing role of interim PET across disease settings but requires confirmation in larger series. In a three-tier evaluation, patients with a DS of 3 behaved similarly to those with a DS of 4–5; we did not adopt this grouping, retaining the conventional DS 1–3 versus 4–5 dichotomy for our primary analysis in keeping with the Lugano framework [5,7], and note the observation only as a signal warranting further study. We found no evidence that marker effects differed by histological subtype, supporting the broad applicability of the DS and the LMR. Consistent with this, the DS-plus-IPS model discriminated well in early-stage disease (C-index 0.88) but only modestly in advanced-stage disease (C-index 0.61), in which the majority of our patients were treated; the model should therefore not be regarded as uniformly applicable across stages, and its overall performance is driven substantially by early-stage patients. Notably, in the advanced-stage subgroup the LMR retained a borderline association with PFS (HR 0.46, 95% CI 0.22–0.99) whereas the DS and IPS did not, an exploratory observation raising the hypothesis that host-derived markers may be comparatively more informative than the interim imaging response in advanced disease.

### 4.1. Strengths and Limitations

The strengths of this study include its real-world, consecutively identified cohort; the use of pre-specified, previously published thresholds to formally test external reproducibility; rigorous internal validation with bootstrap optimism correction; and a transparent distinction between confirmatory and exploratory analyses. This study also has several limitations. It is retrospective, single-center, and modest in size, with 26 PFS events; consequently, subgroup and interaction analyses are underpowered, multivariable models were restricted to two covariates, and the confidence intervals around several estimates are wide. For a two-sided α of 0.05 and 80% power, only moderate-to-large effects were detectable (approximately HR ≥ 3.4 or ≤0.29 for a binary marker, and ≥1.73 per standard deviation for a continuous marker); accordingly, the absence of added value from ΔSUVmax beyond the DS (adjusted HR 0.90, 95% CI 0.78–1.05) should be interpreted as a failure to demonstrate a small incremental effect rather than proof of its absence, and a Type II error cannot be excluded [24]. Overall survival analyses were underpowered given only ten deaths and therefore cannot reliably assess OS prognostic effects. As a single-center, retrospective study it is susceptible to selection bias; because detailed baseline data were not available for patients diagnosed or followed elsewhere, a formal comparison of included and excluded patients was not feasible. The data-derived thresholds—including the exploratory 64–66% ΔSUVmax cutoff and the LMR ≤ 1.82 cutoff—are subject to center-specific overfitting and limited transportability; although corrected for multiplicity and assessed for stability, they should be regarded as exploratory and require external, multicenter validation before clinical use, and our principal LMR analyses were accordingly threshold-independent (based on the continuous variable). All PET/CT reads were performed by a single nuclear medicine physician; a second independent blinded reader was not available, so interobserver reproducibility could not be assessed and the DS and ΔSUVmax were not independently determined. Finally, the histological distribution (predominantly mixed cellularity) differs from that of many Western cohorts and may limit generalizability, although it reflects the real-world population of our region.

### 4.2. Future Directions

Future studies should validate these findings in larger, prospective, multicenter cohorts with harmonized quantitative PET, which would provide the statistical power to determine whether ΔSUVmax offers any incremental value over the Deauville score and to establish reproducible thresholds. External validation of the data-derived LMR cutoff, evaluation of the Deauville-plus-IPS risk model in independent populations, and prospective, stage-stratified assessment—particularly in advanced-stage disease, where interim imaging was less informative—are warranted. Integrating host inflammatory markers with interim imaging and emerging molecular or radiomic biomarkers may further refine early risk stratification in classical Hodgkin lymphoma.

## 5. Conclusions

In this real-world cohort of classical Hodgkin lymphoma, previously published interim ΔSUVmax thresholds were not reproducible in our cohort and require multicenter validation, and quantitative ΔSUVmax did not improve on the visual Deauville score for predicting progression-free survival. In a direct comparison of two candidate refinements of the Deauville score, the host-derived lymphocyte-to-monocyte ratio—but not ΔSUVmax—provided complementary, low-cost prognostic information, consistent with prior reports. A simple, internally validated model combining the interim Deauville score with the International Prognostic Score stratified patients into clinically meaningful risk groups using only routinely available data; external validation is nonetheless required before clinical application. These findings caution against transporting optimized ΔSUVmax thresholds across centers and support the value of inexpensive, host-derived markers alongside interim imaging. Prospective, multicenter validation is warranted before clinical application.

## Figures and Tables

**Figure 1:**
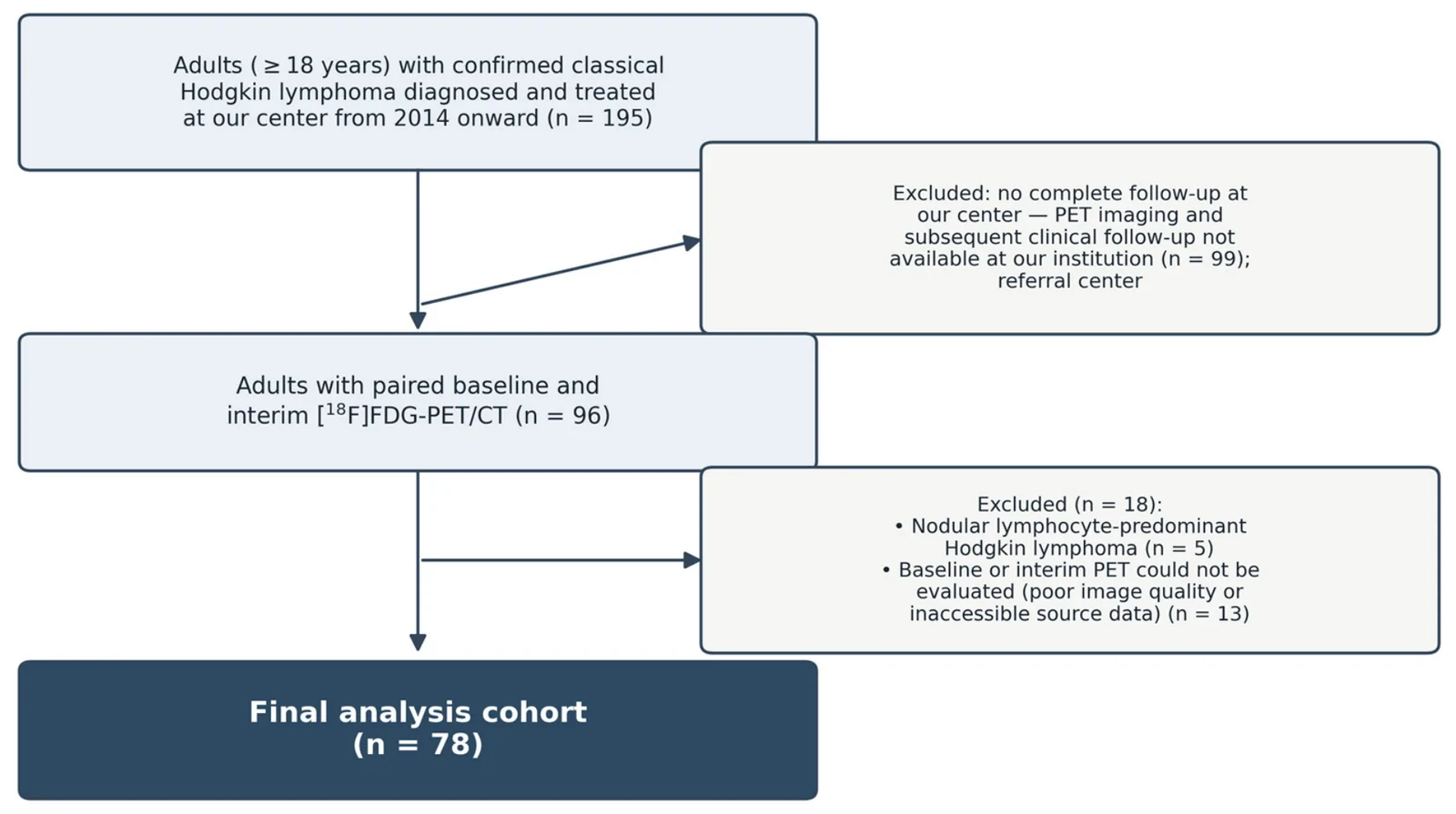
Patient selection flow diagram. Of adults with classical Hodgkin lymphoma diagnosed from 2014 onward, those with paired baseline and interim [18F]FDG-PET/CT were assessed; after exclusions, 78 patients formed the final analysis cohort. Light blue boxes indicate patients retained at each selection step, light gray boxes indicate excluded patients together with the reason for exclusion, and the dark blue box indicates the final analysis cohort; arrows indicate the direction of patient flow.

**Figure 2:**
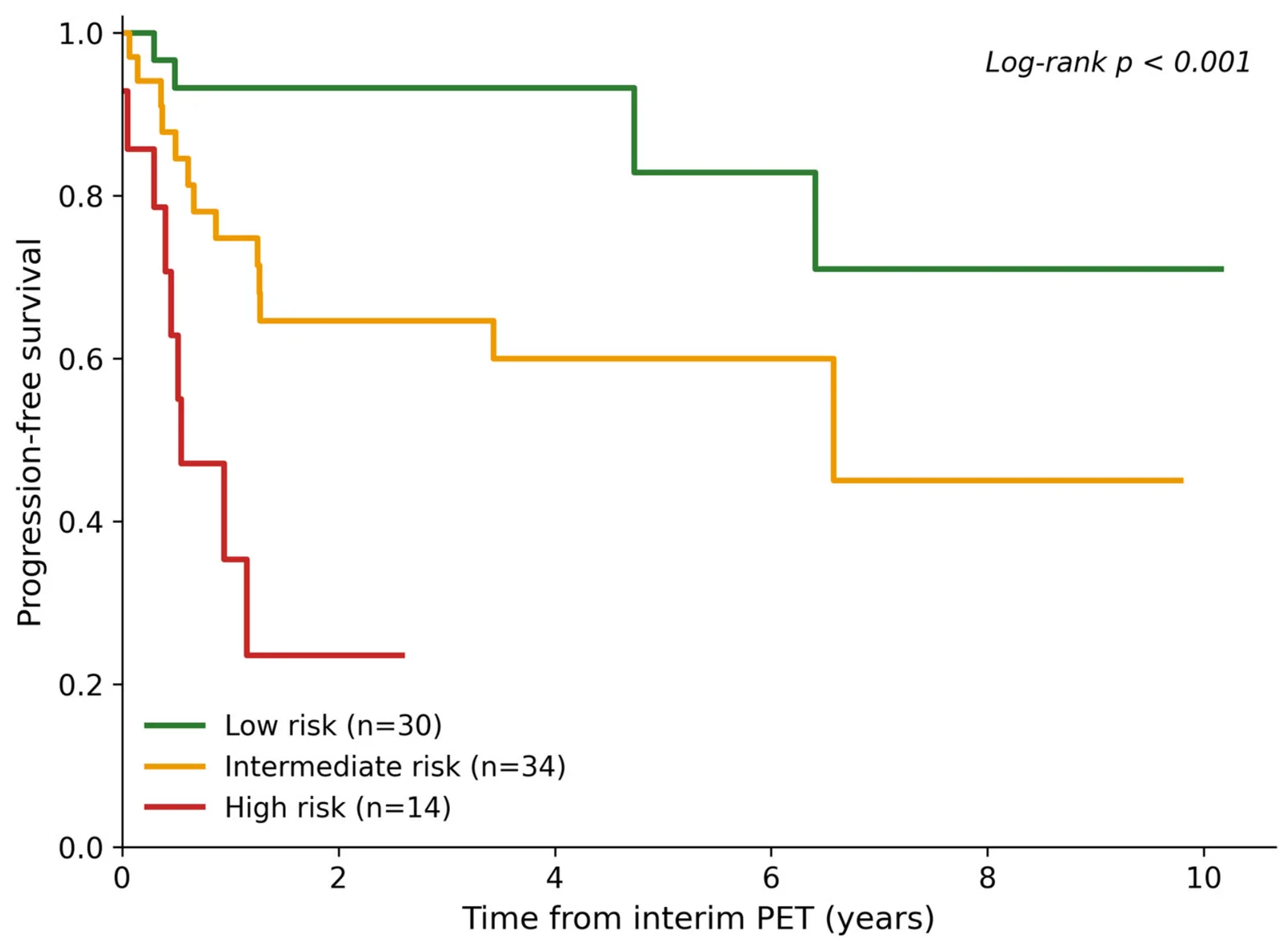
Kaplan–Meier progression-free survival curves for the three-tier Deauville-plus-IPS risk model. Five-year PFS was 82.9%, 60.0%, and 23.6% for the low-, intermediate-, and high-risk groups, respectively (log-rank *p* < 0.001).

**Figure 3:**
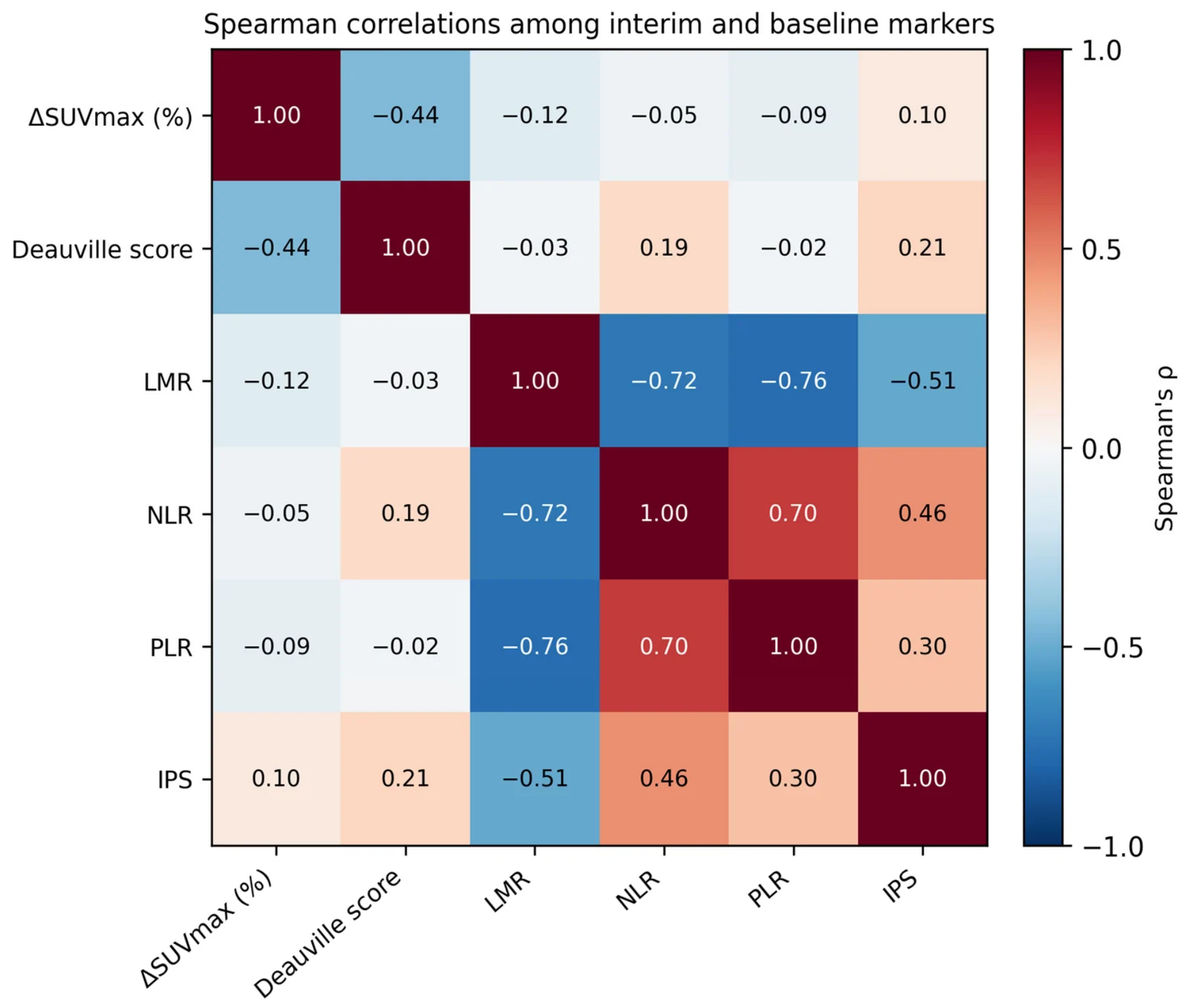
Spearman correlation heatmap among interim and baseline markers. ΔSUVmax correlated moderately with the Deauville score (ρ = −0.44), and the lymphocyte-to-monocyte ratio (LMR) with the International Prognostic Score (ρ = −0.51); the inflammatory ratios were strongly intercorrelated.

**Figure 4:**
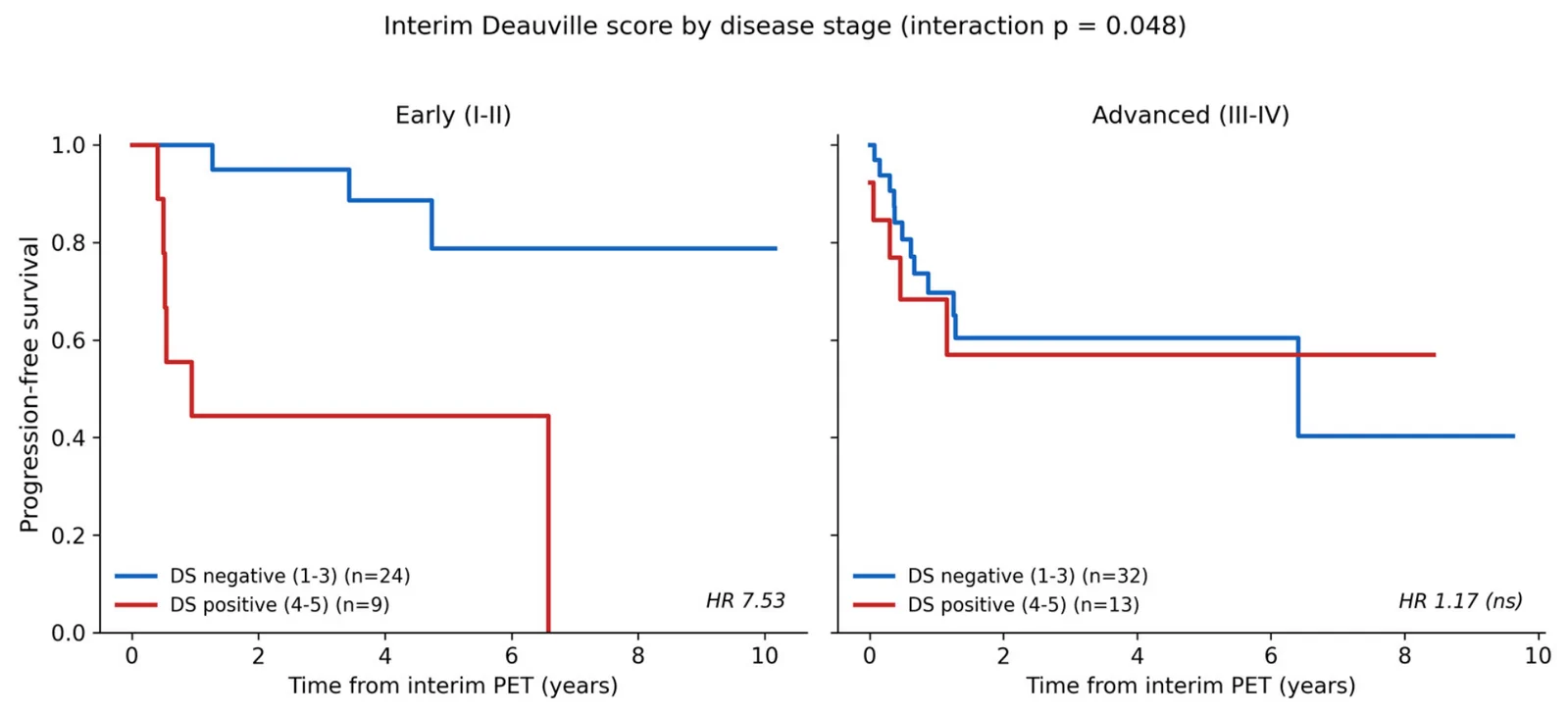
Kaplan–Meier progression-free survival by interim Deauville score, stratified by disease stage. A positive Deauville score was strongly prognostic in early-stage (I–II) disease (HR 7.53) but not in advanced-stage (III–IV) disease (HR 1.17); DS × stage interaction *p* = 0.048.

**Table 1:** Baseline characteristics of the cohort (n = 78).

Characteristic	Value
Age, years, median [IQR]	40.5 [26.5–52.0]
Male/Female, *n* (%)	54 (69)/24 (31)
Histology—Mixed cellularity, *n* (%)	48 (62)
Histology—Nodular sclerosis, *n* (%)	26 (33)
Histology—Lymphocyte-rich, *n* (%)	4 (5)
Ann Arbor stage III–IV, *n* (%)	45 (58)
B symptoms, *n* (%)	46 (59)
Bulky mediastinal disease, *n* (%)	25 (32)
Extranodal involvement, *n* (%)	24 (31)
IPS, median [IQR]	3 [1–4]
ABVD-based first-line therapy, *n* (%)	63 (81)
Interim DS positive (4–5), *n* (%)	22 (28)
Baseline SUVmax, median [IQR]	16.6 [13.0–26.3]
Interim SUVmax, median [IQR]	3.0 [2.2–4.0]
ΔSUVmax (%), median [IQR]	84.0 [73.4–89.7]
LMR, median [IQR]	2.2 [1.2–3.6]
NLR, median [IQR]	4.5 [2.8–6.5]
Hemoglobin, g/dL, mean ± SD	11.5 ± 2.3
Albumin, g/dL, median [IQR]	3.9 [3.5–4.3]
LDH, U/L, median [IQR]	217.5 [178.2–275.0]

IQR, interquartile range; IPS, International Prognostic Score; DS, Deauville score; LMR, lymphocyte-to-monocyte ratio; NLR, neutrophil-to-lymphocyte ratio; LDH, lactate dehydrogenase. Hemoglobin was normally distributed (Shapiro–Wilk *p* > 0.05) and is presented as mean ± SD; all other continuous variables are presented as median [IQR].

**Table 2:** Performance of published ΔSUVmax thresholds and other interim markers for PFS.

Marker/Threshold	Source	5-yr PFS, Favorable vs. Unfavorable	Log-Rank *p*	HR (95% CI)
ΔSUVmax > 66%	Casasnovas/Menton [18,20]	66.3% vs. 45.5%	0.033	2.43 (1.05–5.63)
ΔSUVmax > 71%	Rossi [8]	65.9% vs. 49.9%	0.062	2.19 (0.94–5.07)
ΔSUVmax > 83%	Yang [9]	67.6% vs. 56.4%	0.390	1.40 (0.65–3.02)
ΔSUVmax > 88%	Pinczés [10]	59.3% vs. 63.1%	0.487	0.76 (0.35–1.65)
ΔSUVmax, continuous	—	—	0.024	0.86 per 10% (0.75–0.98)
Deauville positive (4–5)	—	66.9% vs. 51.0%	0.040	2.26 (1.04–4.94)
LMR ≤ 1.82	Data-derived	71.6% vs. 47.0%	0.004	3.13 (1.43–6.84)

HR for the unfavorable group unless otherwise stated. For ΔSUVmax > 93% (Ribeiro), groups were too small to test (favorable n = 3). PFS, progression-free survival; HR, hazard ratio; CI, confidence interval; LMR, lymphocyte-to-monocyte ratio. —, not applicable.

**Table 3:** Discrimination and reclassification of models combining the interim Deauville score with the lymphocyte-to-monocyte ratio (LMR) or the International Prognostic Score (IPS).

Model	C-Index (Apparent)	C-Index (Corrected)	Incremental LR Test
DS alone	0.60	0.60	—
DS + LMR	0.68	0.66	*p* = 0.013 (vs. DS)
DS + IPS	0.75	0.73	*p* < 0.001 (vs. DS)
DS + IPS + LMR	0.75	0.73	—

LMR added to DS + IPS, *p* = 0.34; IPS added to DS + LMR, *p* = 0.015. Reclassification (DS + LMR vs. DS + IPS): NRI −0.52, IDI −0.039. C-index, Harrell’s concordance index (bootstrap optimism-corrected); DS, Deauville score; LR, likelihood-ratio; NRI, net reclassification improvement; IDI, integrated discrimination improvement. —, not applicable.

## Data Availability

The data presented in this study are available on reasonable request from the corresponding author. The data is not publicly available due to privacy restrictions.

## Abbreviations

ABVD	doxorubicin, bleomycin, vinblastine, dacarbazine
CI	confidence interval
cHL	classical Hodgkin lymphoma
DLBCL	diffuse large B-cell lymphoma
DS	Deauville score
[18F]FDG	2-[18F] fluoro-2-deoxy-D-glucose
HR	hazard ratio
IPS	International Prognostic Score
IQR	interquartile range
LMR	lymphocyte-to-monocyte ratio
NLR	neutrophil-to-lymphocyte ratio
OS	overall survival
PET/CT	positron emission tomography/computed tomography
PFS	progression-free survival
PLR	platelet-to-lymphocyte ratio
SUVmax	maximum standardized uptake value
ΔSUVmax	change in maximum standardized uptake value
